# Droplet Epitaxy Image Contrast in Mirror Electron Microscopy

**DOI:** 10.1186/s11671-017-1837-y

**Published:** 2017-01-23

**Authors:** S. M. Kennedy, C. X. Zheng, D. E. Jesson

**Affiliations:** 1 School of Physics, Monash University, Melbourne, Victoria 3800 Australia; 2 Department of Civil Engineering, Monash University, Melbourne, Victoria 3800 Australia; 3 School of Physics and Astronomy, Cardiff University, Cardiff, CF24 3AA UK

**Keywords:** Droplet epitaxy, Gallium arsenide, Mirror electron microscopy, Image simulation

## Abstract

Image simulation methods are applied to interpret mirror electron microscopy (MEM) images obtained from a movie of GaAs droplet epitaxy. Cylindrical symmetry of structures grown by droplet epitaxy is assumed in the simulations which reproduce the main features of the experimental MEM image contrast, demonstrating that droplet epitaxy can be studied in real-time. It is therefore confirmed that an inner ring forms at the droplet contact line and an outer ring (or skirt) occurs outside the droplet periphery. We believe that MEM combined with image simulations will be increasingly used to study the formation and growth of quantum structures.

## Background

The self-assembly of semiconductor nanostructures has received significant interest because of potential applications in nanoscale optoelectronics and quantum information technologies [1–4]. Droplet epitaxy has recently emerged as a flexible technique for tailoring the morphology of quantum structures [3, 5–11] including dots, double-dots [5], molecules [6], rings [7] and double-rings [8, 9]. In this approach, typically group-III liquid metal droplets are first deposited on a semiconductor surface such as GaAs. Then exposure to a group-V flux results in the formation of a crystalline epitaxial quantum structure. Clearly, it is desirable to develop and apply techniques to study the processes of droplet epitaxy in real-time in order to understand basic growth mechanisms and optimise the control of quantum structure morphology for potential device applications.

Mirror electron microscopy (MEM) is a well-established technique for imaging surface structures and potentials [12–19]. In this approach, an electron plane wave is directed at a negatively charged specimen such that the electrons reverse in direction above the sample surface. The electrons are then reaccelerated in the low energy electron microscope column before being directed to the imaging system. In the turn-around region, the electrons are sensitive to variations in electric field which can be produced by changes in surface height or work function across the specimen surface. This results in the deflection of electrons, which redistributes their positions on the detector, producing image contrast.

MEM has a number of advantages for the study of nanostructure formation. Since the electron beam does not impact the surface, it is a non-destructive technique which can be applied to study sensitive specimens. Furthermore, the parallel nature of the technique facilitates the acquisition of real-time movies of surface evolution, permitting dynamic studies of droplet epitaxy [20] and dynamics [21–23]. Despite these important advantages, MEM image contrast can be highly non-intuitive since it arises from electric or magnetic field variations above the specimen. In the special case of weak electron deflections, the images can be interpreted using Laplacian imaging theory [24, 25]. However, in general, for larger deflection of electrons, such as those arising from liquid droplets or quantum structures, the images consist of envelopes of electron rays or caustics. While such caustics contribute to the non-intuitive nature of MEM images, they can be simulated using a recently developed caustic imaging theory [26]. The purpose of this paper is to investigate whether caustic imaging theory can be used to interpret image contrast arising during droplet epitaxy in terms of surface morphological evolution.

## Methods

**Fig. 1:**
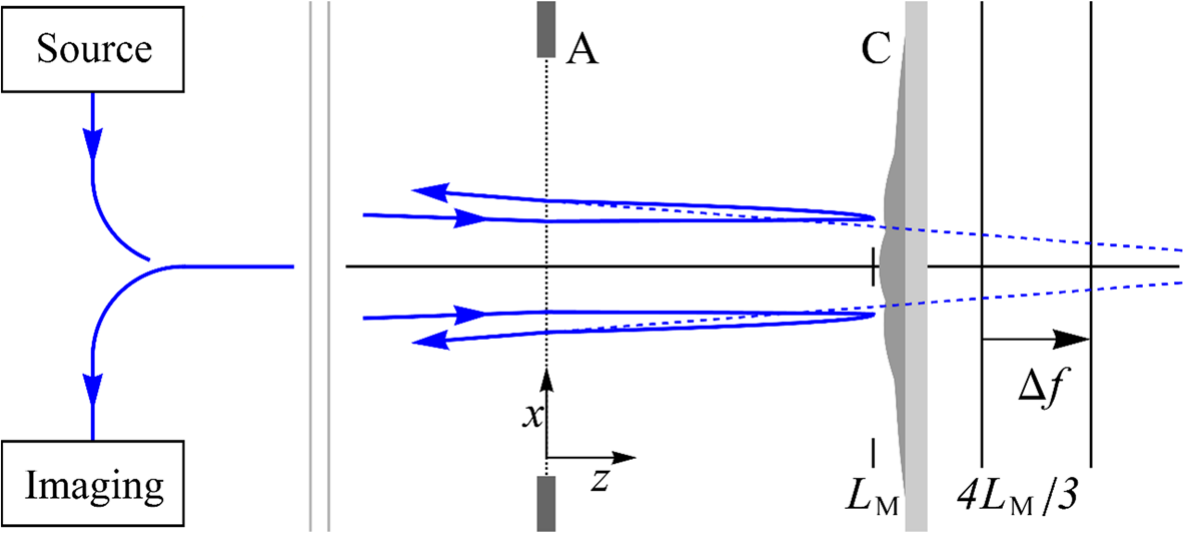
MEM imaging geometry. The electron beam (*blue line*) travels along the *z*-axis. The beam is converging and is focused towards the point $\left(x=0,\;y=0,\;z=4{L}_{\mathrm{M}}\right)$. The beam passes through an aperture in the grounded anode $\mathrm{A}\;\left(z=0\right)$ and is deflected slightly as the beam enters the electric field in the region $0\le z\le L$, where the cathode $\mathrm{C}$ is at $z=L$. This deflection causes the beam to travel parallel to the $z$-axis, resulting in parallel illumination of the sample. The electron beam turns around in the vicinity of the turning distance $z={L}_M=L-\delta$, for some small distance $\delta$. After interacting with the electric field above the cathode surface (held at a potential of $V$) in the vicinity of $z={L}_M$, the deflected electron beam is reaccelerated away from the cathode and passes back through the anode aperture $A$ $\left(z=0\right)$. The microscope is assumed to form an image of the electron positions as they would appear on a virtual image plane at $z=\Delta f+4{L}_M/3$. Here, $\Delta f$ is the defocus distance from the plane $z=4{L}_M/3$ and is controlled by the magnetic part of the objective lens. The $y$-axis extends out of the page

The imaging geometry associated with MEM is shown schematically in Fig. 1 where a converging electron beam of energy *U* passes through a grounded anode aperture A and emerges parallel to the optical axis *z*. A quantum structure specimen is located at *z* = *L* and forms the cathode of the immersion objective lens. This is held at a negative potential *V* by applying a small voltage relative to the grounded anode. This potential is sufficiently negative (< − *U*/*e*) to cause the electron beam to reverse in direction at *z* = *L*_M_, a distance *δ* from the cathode surface such that

1 $$ {L}_{\mathrm{M}}=L-\delta =- LU/ eV, $$

where − *e* is the electronic charge. Following deflection by the electric field surrounding the quantum structure surface, the electron beam is then reaccelerated into the imaging system of the microscope. The MEM image results from the redistribution of electrons on the virtual image plane at *z* = *Δf* + 4*L*_m_/3 where the defocus distance *Δf* is controlled by the magnetic part of the objective lens.

**Fig. 2:**
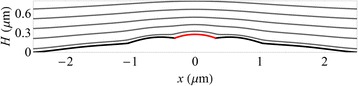
Equipotential surfaces. The equipotential surfaces of the electric potential (*grey lines*) above a surface that is rotationally symmetric about the $x=0$ axis, with a cross section in the $x\hbox{--} z$ plane shown. The surface and cathode are kept at a potential of $V=-20,000.4$ V, and the *red region* corresponds to the remnant liquid Ga droplet which has a work function of $-0.3$V compared to the rest of the surface. The first equipotential line above the surface is at $-19,999.9$ V, and the subsequent lines are +1.5 V apart (e.g. the second is at $-19,998.4$V)

To simulate the image contrast resulting from the morphologies arising during droplet epitaxy, we must first evaluate the electric potential in the vicinity of typical quantum structures. This is accomplished by solving Laplace’s equation using finite element methods with the specimen topography as one boundary and the grounded anode as the opposite boundary [26]. We utilise the FreeFEM++ finite element package with 300,000 mesh points in total and mesh adaptation [27] which reduces the mesh size where the electric potential changes more rapidly. For simplicity, we approximate the quantum structure as cylindrically symmetric so we need only evaluate the electric potential in two dimensions (2D). Then the three dimensional (3D) electric potential above the structure surface can be generated from a 2D height profile slice. This is illustrated in Fig. 2 which shows the equipotential surfaces for a 2D height profile for the experimental parameters *U* = 20 keV, *V* = −20,000.4 V and *L* = 2 mm. The full 3D cylindrically symmetric electric potential can be obtained by simply rotating the equipotential lines about *x* = 0.

To generate the MEM image intensity, a family of electron ray trajectories is traced through the electric potential using a fourth-order Runge-Kutta method [26, 28]. The incident electron paths begin at *z* = 0 with an equal spacing of rays *x*_0_ (=10 nm), along the *x*-axis, i.e. the vertical axis of Fig. 1. These initially parallel rays are traced through the turn-around region in the vicinity of the quantum structure and back to the anode aperture. The emerging rays are then projected back to the virtual image plane at *z* = *Δf* + 4*L*_m_/3 as shown in Fig. 1. The MEM image intensity *I*(*x*, *δ*, *Δf*) is then evaluated in this plane by comparing the distance between two initially adjacent rays *s*(*x*, *δ*, *Δf*), with the equal spacing expected for an equipotential flat specimen giving

2 $$ I\left(x,\delta, \varDelta f\right)=\frac{x_0\left(\frac{2}{3}-\frac{\varDelta f}{4{L}_{\mathrm{m}}}\right)}{s\left(x,\delta, \varDelta f\right)}. $$

The image intensity may then be expressed as a 2D plot by exploiting the cylindrical symmetry. Where initially adjacent rays cross (i.e. *s*(*x*, *δ*, *Δf*) → 0) caustics are formed in the image. This can be treated numerically by choosing a threshold ray spacing below which we assign a fixed value to *I*(*x*, *δ*, *Δf*) in Eq. (2). This is equivalent to specifying the saturation level of the detector. A work function difference of –0.3 V between liquid Ga and GaAs is applied to the simulations when liquid is part of the quantum structure [26, 29].

**Fig. 3:**
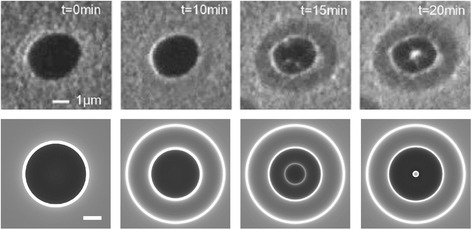
Comparison of experimental and simulated MEM contrast. (*Top row*) sequence of experimental images of various stages of droplet epitaxy at the indicated times [20]. (*Bottom row*) sequence of simulated MEM images at a defocus of $\Delta f=-42\;\upmu \mathrm{m}$, electron initial energy $U=20$ keV, *L* = 2 mm, cathode potential of $V=-20,000.4$V and liquid Ga work function difference of −0.3 V. The simulations used 1201 rays with initial spacing ${x}_0=10$ nm, with the intensity calculated using Eq. 2. The height profiles used for each of the four time steps are shown in Fig. 5 and were based on AFM traces of representative stages of droplet epitaxy (Fig. 4)

To investigate the applicability of caustic imaging theory to interpret MEM movies of droplet epitaxy, we examine a time-sequence of images which has previously been used to deduce mechanisms of ring formation [20]. It is therefore important to test and verify these conclusions via image simulation. The movies were obtained using a LEEM specifically designed for III–V epitaxy [30]. An undoped GaAs (001) epi-ready wafer was degassed at 300 °C under ultrahigh vacuum for 24 h. This was followed by high temperature flashing up to 600 °C and annealing at 580 °C for 2 h to remove the surface oxide. Ga droplets were then prepared by annealing above the congruent evaporation temperature at 650 °C. The sample temperature was reduced to 460 °C and images were recorded in MEM mode [12–19, 26]. The As shutter was opened at *t* = 0 min, exposing the Ga droplet to an As_4_ flux beam equivalent pressure (BEP) 1.45 × 10^−5^ Torr. Snapshots from a resulting MEM movie of droplet crystallization are shown in Fig. 3 [20].

**Fig. 4:**
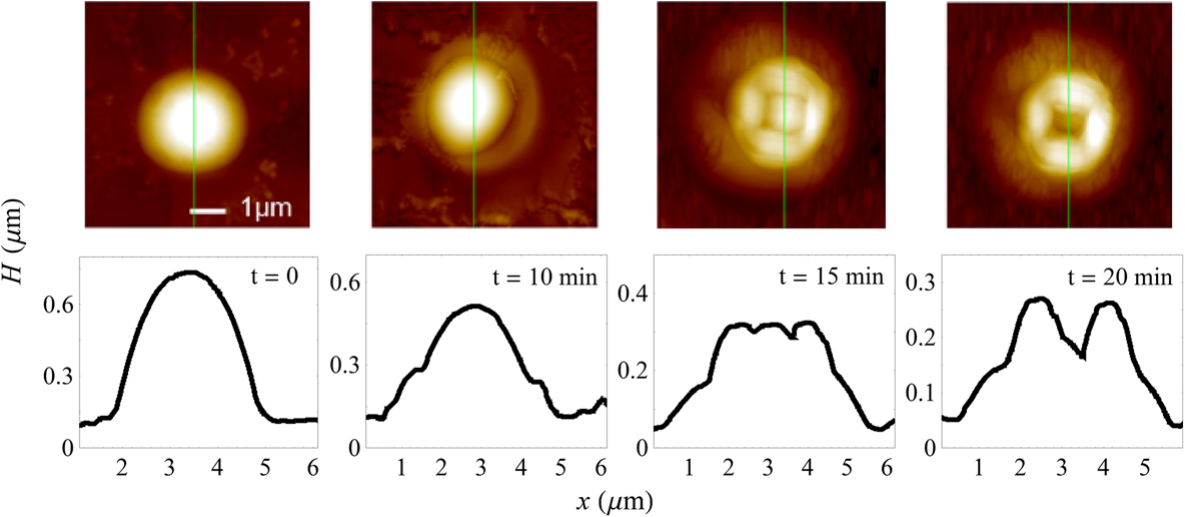
AFM of representative stages of droplet epitaxy [20]. The top row displays AFM images. Associated cross sections corresponding to the green line traces are shown below each image

To assist in the interpretation of MEM contrast, additional experiments were performed in which Ga droplets were exposed to As_4_ at identical flux and temperature for fixed times of 10, 15 and 20 min, at which point they were quenched to room temperature. The samples were then examined by atomic force microscopy (AFM) and typical observed morphologies are contained in Fig. 4.

## Results and Discussion

**Fig. 5:**
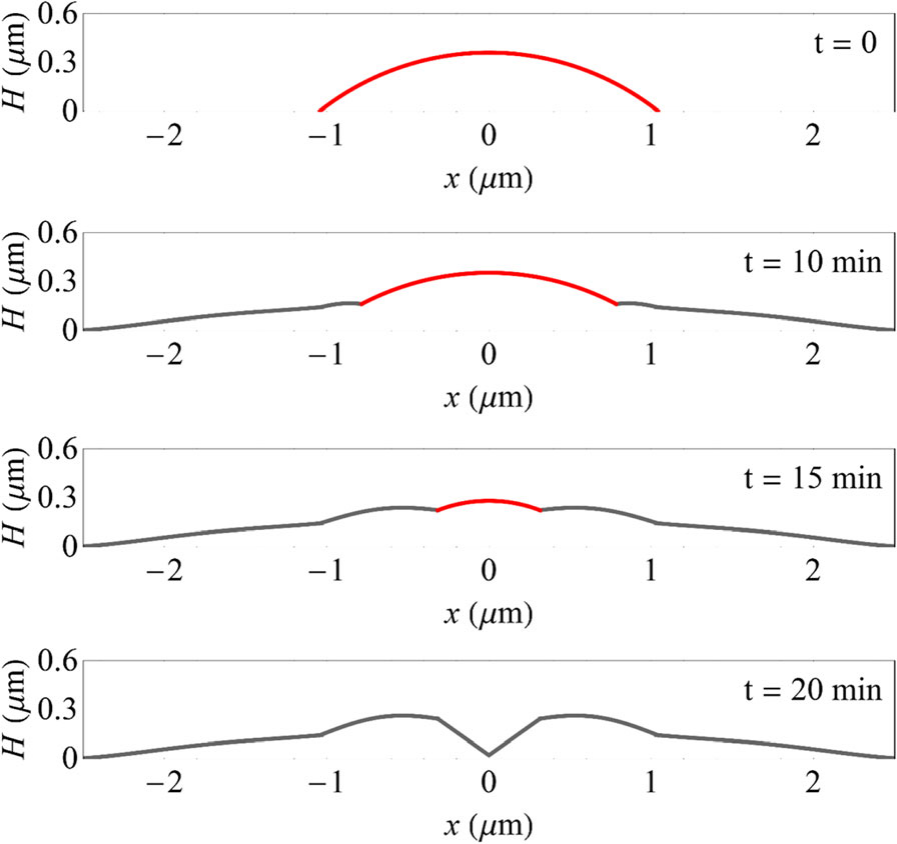
Height profiles used in the MEM simulations of droplet epitaxy. These are displayed for $t=$ 0, 10, 15 and 20 min. The AFM traces of Fig. 4 were used to guide the shape of these model profiles. The *red regions* indicate the liquid Ga droplet or its remnant

The AFM data contained in Fig. 4 can only be used as an approximate guide to the surface shape under actual growth conditions. The data was obtained from different droplets and so does not represent a time evolution of a single quantum structure. Furthermore, quenching to room temperature may induce artefacts and the observed morphologies may not exactly reflect the shapes undergoing droplet epitaxy at 460 °C. Nevertheless by appropriate scaling of the features, the AFM data can be used as an approximate guide to the surface morphology. Using profiles generated from this data as an initial starting point, we have performed MEM image simulations and further fine-tuned the surface features iteratively to obtain a best fit to the experimental data in Fig. 3. The resulting profiles are shown in Fig. 5 which generate the MEM image simulations displayed in Fig. 3.

**Fig. 6:**
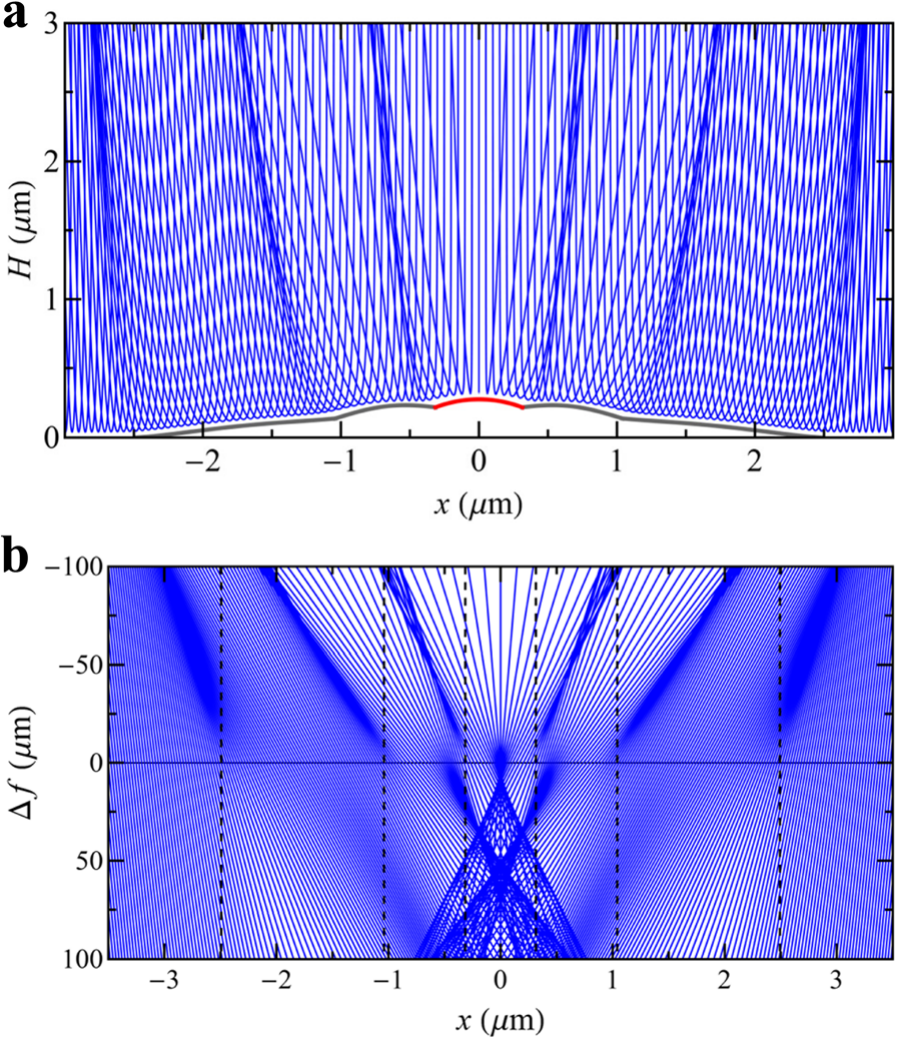
The family of electron rays simulated in the MEM system of Fig. 1, incident on the $t=15$ min surface as shown in Figs. 2 and 5. **a** Electron paths close to the surface demonstrate how changes in the height, e.g. discontinuities, can cause electron paths to overlap in the returning beam. **b** Apparent straight line paths of the exiting electrons traced back to the virtual image plane $z=\Delta f+4{L}_{\mathrm{M}}/3,$ for defocus $\Delta f$ controlled by the magnetic part of the objective lens. The $x$ positions of the discontinuities in the surface height profile are indicated by *vertical dashed lines*. The $x$ positions of the rays have been multiplied by $3/2$ to remove the demagnification of the anode aperture as discussed in [26]. Note that the changes in the surface height function create regions where electron paths overlap (both in the returning beam and near the virtual imaging plane), which are evident as bright caustic rings in the simulated image (Fig. 3)

It can be seen from Fig. 3 that the simulations reproduce the salient features of the images. In general, bright rings are associated with discontinuities in the surface profiles in Fig. 5. This can be explained by studying how the electron rays are deflected by the morphology at *t* = 15 min as shown in Fig. 6a. Changes in the surface height profile, e.g. discontinuities, create subsequent changes to the equipotential surfaces above the surface (Fig. 2). Although it can be seen that the equipotential surfaces somewhat smooth the surface discontinuities, electron trajectories at either side of these discontinuities are deflected in different directions which causes electron paths to overlap in the returning beam (Fig. 6a). A projection of these emerging rays back to the virtual image plane at *z* = *Δf* + 4*L*_m_/3 results in the overlapping trajectories shown in Fig. 6b which creates bright caustic features in the images. Hence, the existence of bright caustic rings observed in the image (Fig. 3) can be directly related to the discontinuities in the surface profile. Note that the positions of the surface discontinuities and caustics are similar at small defocus, but diverge with increasing *Δf* (or work function difference) (Fig. 6b).

**Fig. 7:**
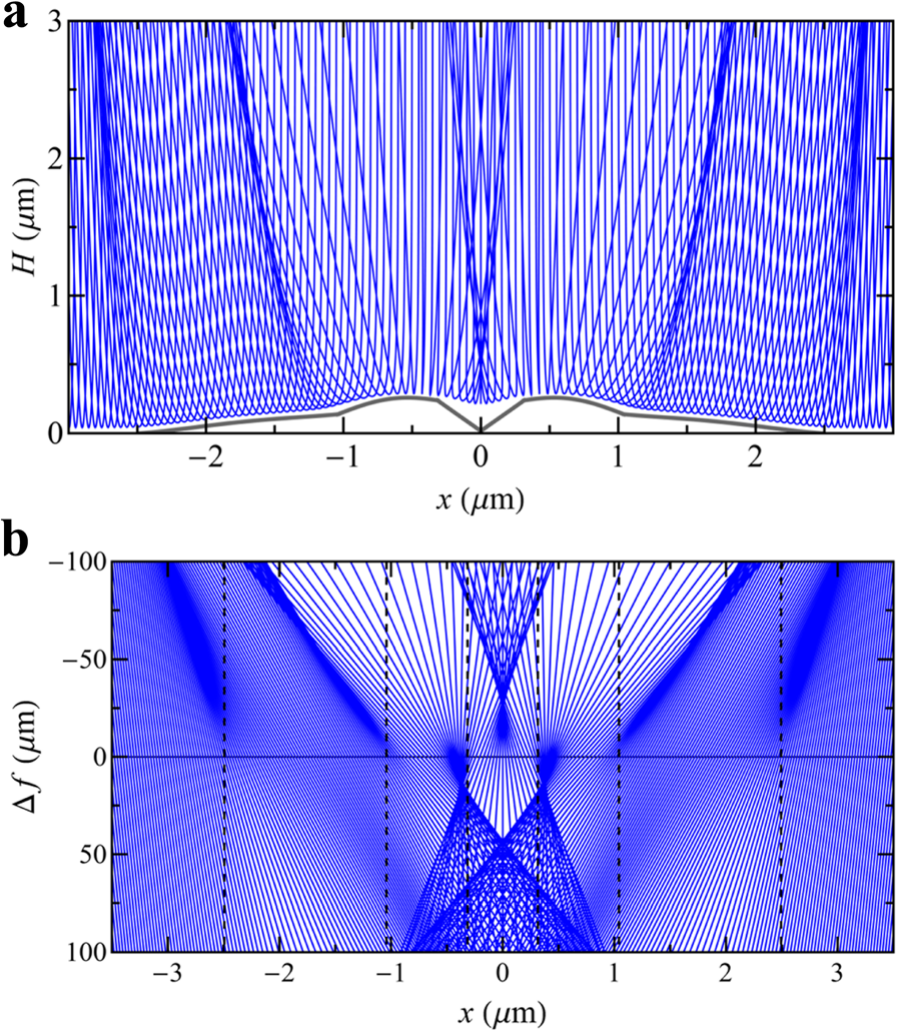
The family of electron rays simulated in the MEM system of Fig. 1, incident on the $t=20$ min surface as shown in Fig. 5. **a** Electron paths close to the surface demonstrate how the surface can act as an electron lens to focus the electrons in the returning beam. **b** Apparent straight line paths of the exiting electrons traced back to the virtual image plane $z=\Delta f+4{L}_M/3,$ for defocus $\Delta f$ controlled by the magnetic part of the objective lens. The $x$ positions of the discontinuities in the surface height profile are indicated by *vertical dashed lines*. The $x$ positions of the rays have been multiplied by $3/2$ to remove the demagnification of the anode aperture as discussed in [26]. Note that the focusing of the electron beam in the returning trajectories creates a bright caustic central spot on the virtual image plane and the simulated image (Fig. 3)

Although the image simulations in Fig. 3 reproduce the main contrast in the MEM images, there are some minor discrepancies worthy of discussion. For example, the outer bright concentric ring present in the simulations is not as pronounced in the images. This is probably due to intrinsic roughness of the surface in this region which lowers the experimental contrast. A second feature is that the experimental images at *t* = 15, 20 min both display approximately fourfold symmetry towards the image centre. This is clearly linked to surface energy anisotropy and faceting in the case of the *t* = 20 min central crater. Clearly, this cannot be reproduced by the cylindrical symmetry of our simulations and would require a full 3D simulation. Nevertheless, our simulations capture the appearance of a central bright spot at *t* = 20 min which reflects the crater acting as an electron lens and focussing the electrons to a caustic (Fig. 7).

Effects of spherical aberration can be incorporated into the simulations by adding appropriate shifts to the position of rays in the virtual image plane [26]. Similarly, chromatic aberration can be included by taking a weighted average of a series of monochromatic intensity images for a spread of energy values [26]. However, for spherical aberration coefficient *C*_s_ ≈ 0.1 m and a Gaussian energy spread of full-width half-maximum 0.3 eV, we find both aberrations have a negligible effect for the relatively low resolution case considered here.

The ability to simulate the surface profile and compare with experiment allows us to deduce several important features regarding the mechanisms of droplet epitaxy [20]. Firstly, as can be seen at *t* = 10 min, deposition of a GaAs inner ring starts to occur at the original position of the droplet contact line. This has been interpreted in terms of enhanced material deposition at the contact line as a result of the vertical force exerted on the substrate in this region [31]. Secondly, the outer ring or skirt forms immediately outside of the droplet periphery indicating it is due to the reaction of Ga adatoms diffusing away from the droplet with deposited As flux. These key observations have been used as a basis for a theory of droplet epitaxy which can explain all of the experimentally observed quantum structures obtained using this technique [20]. The simulations presented here confirm this interpretation.

## Conclusions

We have demonstrated, through MEM image simulations, that the time evolution of the surface profile can be determined during droplet epitaxy in real-time. This has confirmed that an inner ring forms at the droplet contact line and an outer ring (or skirt) occurs outside the droplet periphery. These are valuable observations for creating a theory of droplet epitaxy. We believe that the use of MEM combined with image simulations will be used more generally to study the growth and fabrication of quantum structures. This can be achieved at higher resolutions as required, but will likely require the inclusion of spherical and chromatic aberration into the simulation methods to model image contrast.
